# Case Report: Novel MFSD8 Variants in a Chinese Family With Neuronal Ceroid Lipofuscinoses 7

**DOI:** 10.3389/fgene.2022.807515

**Published:** 2022-01-26

**Authors:** Yimeng Qiao, Yang Gu, Ye Cheng, Yu Su, Nan Lv, Qing Shang, Qinghe Xing

**Affiliations:** ^1^ Institutes of Biomedical Sciences and Children’s Hospital, Fudan University, Shanghai, China; ^2^ Children’s Hospital of Zhengzhou University and Henan Children’s Hospital, Zhengzhou, China; ^3^ Shanghai Center for Women and Children’s Health, Shanghai, China

**Keywords:** neuronal ceroid lipofuscinoses, CLN7, MFSD8, whole gene deletion, mutation

## Abstract

Neuronal ceroid lipofuscinoses (NCLs) are among the most common progressive encephalopathies of childhood. Neuronal ceroid lipofuscinosis 7 (CLN7), one of the late infantile-onset NCLs, is an autosomal recessive disorder caused by mutations in the *MFSD8* gene on chromosome 4q28. Almost all reported mutations of *MFSD8* in CLN7 patients were SNVs. However, we report a 4-year-old boy with CLN7 harboring compound heterozygous mutations in the *MFSD8* gene, including one novel two-nucleotide deletion c.136_137delAT (p. M46Vfs*22) and one whole gene deletion of *MFSD8* confirmed by Sanger sequencing, genomic quantitative PCR and CNV-seq. Therefore, for nonconsanguineous CLN7 patients with homozygous mutations in the *MFSD8* gene, genetic counseling staff should focus on the possibility of whole gene deletion. This is one case report describing a whole gene deletion in a Chinese patient with CLN7, suggesting the diagnosis of CLN7 should be based on clinical suspicion and genetic testing.

## Introduction

Neuronal ceroid lipofuscinoses (NCLs), among the most common progressive encephalopathies of childhood, with ages of onset ranging from birth to adulthood (Williams et al., 2006; Jalanko and Braulke, 2009), are a clinically and genetically heterogeneous group of neurodegenerative disorders (Schulz et al., 2013; Zare-Abdollahi et al., 2019). NCLs are ultrastructurally characterized by the intracellular accumulation of autofluorescent lipopigment storage material in different patterns (Goebel and Wisniewski, 2004). Patients developing such disorders may suffer from cognitive impairment (HP:0100543), motor deterioration (HP:0002333), progressive visual loss (HP:0000529), and uncontrolled seizures (HP:0001250) (Goebel and Wisniewski, 2004; Mole et al., 2005). NCLs are historically classified into four major subtypes based on the age of onset: infantile, late infantile, juvenile, and adult (Butz et al., 2020). To date, thirteen causal genes have been identified in different subtypes (Kohan et al., 2011; Chartier et al., 2021). One gene can be involved in different clinical forms, and conversely, one clinical form can be due to different genes (Mole et al., 2019).

Neuronal ceroid lipofuscinosis 7 (CLN7; OMIM: 610951) is an autosomal recessive disorder caused by mutations in the *MFSD8* gene on chromosome 4q28 (Kousi et al., 2009). *MFSD8* (NP_001358525) encodes a 518-amino acid integral lysosomal transmembrane protein that includes twelve membrane-spanning domains with cytosolic N- and C-terminal domains (Siintola et al., 2007). More than 40 different *MFSD8* mutations, including missense, nonsense or Indel mutations, have been detected, causing an accumulation of cellular glycoproteins and lipoproteins, ultimately resulting in cellular degeneration and the occurrence of CLN7 (McBride et al., 2018).

To date, next-generation sequencing (NGS), which enables fast and cost-effective generation of genome-scale sequence data with exquisite resolution and accuracy, has been increasingly used as a diagnostic tool in the clinic (Xuan et al., 2013; Sawyer et al., 2016). Technologies including target NGS panel (Panel), clinical exome sequencing (CES), whole exome sequencing (WES), CNV-seq and whole genome sequencing (WGS) have greatly improved the diagnosis rate for inherited diseases (Xue et al., 2015; Monlong et al., 2018).

We report one case of a 4-year-old boy with CLN7 who presented with global developmental delay (GDD) (HP:0001263), sleeping disturbance (HP:0002360), and ataxia (HP:0001251) with no ocular abnormalities (HP:0000478) and who harbored novel compound heterozygous mutations in *MFSD8* (c.136_137delAT, p. M46Vfs*22, inherited from the mother and one whole gene deletion, inherited from the father). This is the first report of two severe *MFSD8* trans-mutations associated with CLN7 in a Chinese family.

## Methods and Materials

### Subjects and Clinical Assessment

Four members of a nonconsanguineous Chinese family with one individual affected by GDD were recruited for this study. Magnetic resonance imaging (MRI) and the 24-h video electroencephalogram (EEG) were performed on the proband and an additional three non-affected family members. The Psychiatric Class B scale and the Sign-significant relations were used to assess the developmental characteristics of the proband. After obtaining written informed consent, peripheral venous blood samples were collected from the four family members. A statement of informed consent was obtained from the parents of all subjects under the age of 18 after full explanation of the procedure. The research protocol was reviewed and approved by the ethics committee of Henan Children’s Hospital in accordance with the Declaration of Helsinki.

### Whole Exome Sequencing and CNV-seq

Total genomic DNA was extracted from peripheral blood with the QIAamp^®^ DNA Blood Mini Kit (QIAGEN, Hilden, Germany) according to the manufacturer’s protocol. A NanoDrop spectrophotometer was used to measure DNA purity and concentration. The method of WES and CNV-seq was previously described (Gao et al., 2019).

For WES-seq, DNA libraries were prepared using xGen Exome Research Panel v1.0 (Integrated DNA Technologies, Inc., Coralville, IA, United States) according to manufacturer’s protocol. Sequencing was performed on Illumina HiSeq2500 platform to a mean per-sample depth of 100×. After sequencing, the reads were mapped to the hg19 human reference genome using Burrows-Wheeler Aligner and variants were called using the Genome Analysis Toolkit (GATK). SAMtools and Pindel were subjected to call SNVs and Indels, respectively. The variants with minor allele frequency >1% in dbSNP, NHLBI exome sequencing project (ESP1), ExAC database, the 1,000 genomes project database, and 200 in-house controls were removed. The pathogenicity of the variants was predicted using the Sorting Intolerant From Tolerant (SIFT), Polymorphism Phenotyping v2 (PolyPhen-2), Combined Annotation Dependent Depletion (CADD) and Mutation Taster. Each variant identified in a known neurodevelopmental disease gene as selected from OMIM (4 August 2021) could be considered as potential disease-causing variants according to the guidelines of the American College of Medical Genetics and Genomics (ACMG). Possible pathogenic genes were identified based on heredity models, deleterious variants and clinical phenotypes.

For CNV-seq, more than 1 µg genomic DNA was broken into 200–300 bp fragments for library preparation and sequenced on the Illumina HiSeq 2,500 (Illumina, San Diego, United States). Raw data were analyzed by fastp v0.18.1 software, and clean data were then subjected to human reference genome (hg19) analysis using BWA. After PCR duplications were removed by Picard MarkDuplicates, the mixture-hidden Markov model (*m*-HMM) approach was applied to estimate the window-based copy number change points and copy number states. An in-house pipeline was used to map and call CNVs larger than 100 kb. Candidate CNVs were annotated by analyzing the genes contained in the CNVs and the CNV intervals themselves with the databases Decipher v11.8, ClinVar, ClinGen, and OMIM. Then the candidate CNVs were filtered with normal frequency databases DGV v107 and ISCA. Interpretation of constitutional CNV was based on annotation information and frequency database (less than 0.5%) according to ACMG as reported previously.

### Sanger Sequencing

The candidate disease-causing variants were confirmed by Sanger sequencing. Amplification was performed using the following primers: 5′-GTT​CTA​GGG​ATT​GGT​TGC​ACA -3′ (forward) and 5′- TGC​TGT​CAT​CAA​GTA​GAG​CAT​G-3′ (reverse). Parent and sibling samples, where available, were also analyzed by Sanger sequencing to determine if the origin of the variant was paternal, maternal, or *de novo*.

### Genomic Quantitative PCR

Deletions in *MFSD8* were validated by qPCR using ABI Quant Studio six Flex (Thermo Fisher). We designed two pairs of primers within the *MFSD8* gene (Supplementary Table S1). qPCR was carried out in a total volume of 10 μL containing 5 μL of TB Green^®^ Premix Ex Taq™ (Tli RNaseH Plus) (TaKaRa Biotechnology, Dalian, China), 0.2 μL ROX Reference Dye II, 0.4 μM of each primer and 20 ng of genomic DNA. All samples were run in triplicate. Thermal cycling conditions were 95°C for 30 s, followed by 40 cycles at 95°C for 5 s and 60°C for 30 s. The *ALB* gene was selected as the control amplicon. The dosage of each amplicon relative to *ALB* and normalized to the mother’s DNA was determined using the 2^−ΔΔCt^ method.

## Results

### Case Description

**FIGURE 1:**
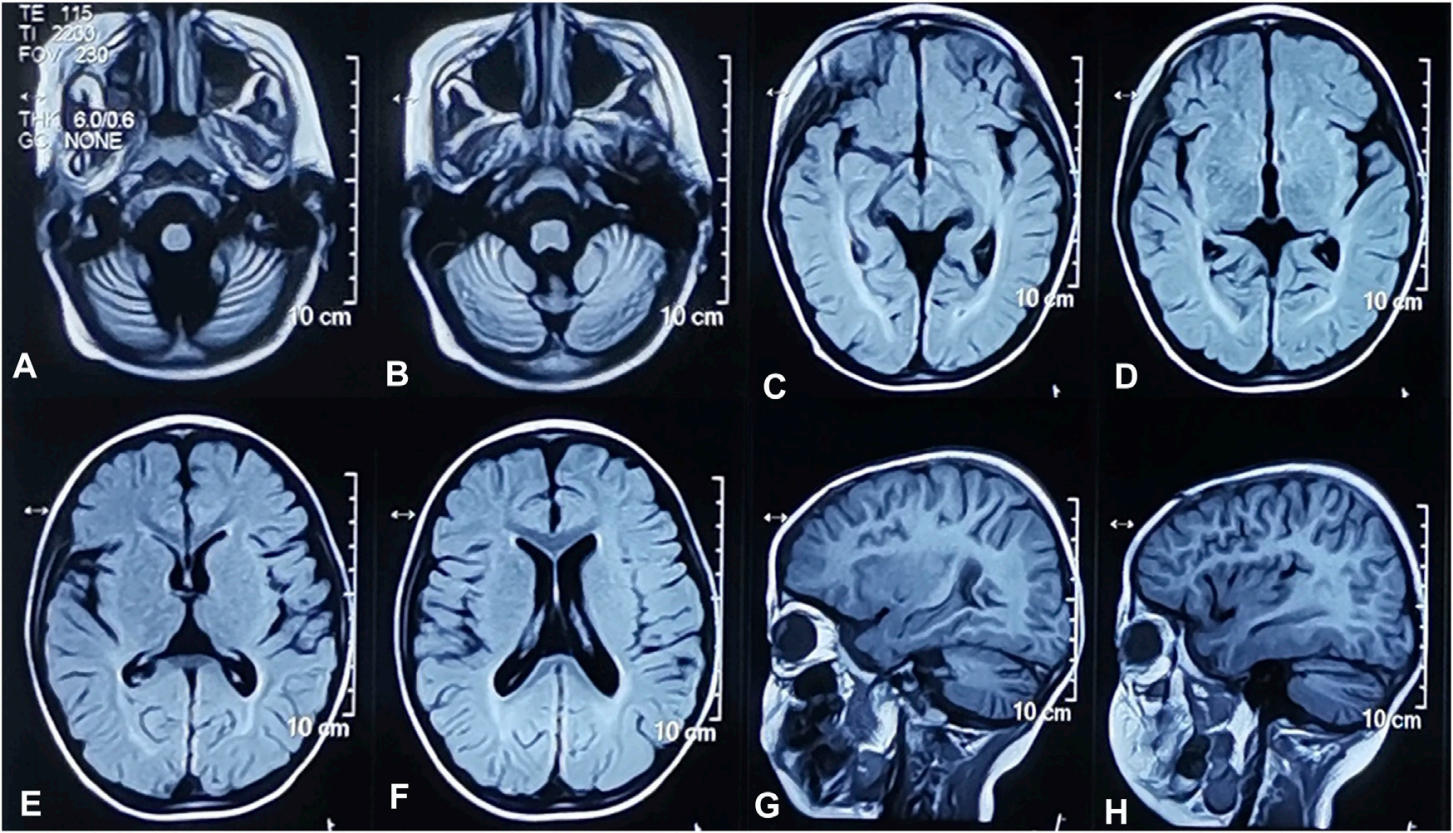
Brain MRI of the proband. Widened cerebellum sulci. Axial T2-weighted FLAIR image **(A,B)**. Abnormal signals of white matter near the posterior horn of bilateral lateral ventricles. Axial T2-weighted FLAIR images **(C,D)**. Widened bilateral cerebral hemispheres sulci. Axial T2-weighted FLAIR image **(E,F)**. Widened bilateral cerebral hemispheres and cerebellar sulci. Sagittal T1-weighted images **(G,H)**.

The proband, a 4-year-old boy, was born after an uneventful pregnancy and delivery, with nonconsanguineous marriage of his parents. He was born at the gestation period of 36 weeks plus 5 days with a birth weight of 3,500 g. The patient suffered jaundice approximately 1 month after birth and received blue light radiation and oral medication. He presented some phenotypes, such as cognitive impairment, motor deterioration, expressive language delay (HP:0002474), sleeping disturbance, and ataxia (HP:0001251), with no ocular abnormalities. His lower limbs were prone to involuntary tremors and he would fall when tired or frightened. On physical and neurological examinations at admission, he had a weight of 18.3 kg, a height of 105 cm, and a head circumference of 49.5 cm. The MRI of the brain showed bilateral widening of the cerebral hemispheres and cerebellar sulci and abnormal white matter signals in the posterior horns of both sides of the ventricle (Figure 1). The 24-h video EEG showed abnormal activity: widespread sharp waves, sharp slow waves, and pointed slow wave emission. Slow waves were discharged from the tip of his right forehead during sleep. According to the Psychiatric Class B scale, at the age of four, his exercise and social adaptation were equivalent to 12 months, and his intelligence was equivalent to only 11 months. The development score showed the mental index (MI) < 44 points and the developmental quotient (DQ) < 44 points. The Sign-significant relation (S-S) also indicated language developmental delay. Due to these clinical and laboratory findings, he was diagnosed with GDD.

### Genetic Analyses

**FIGURE 2:**
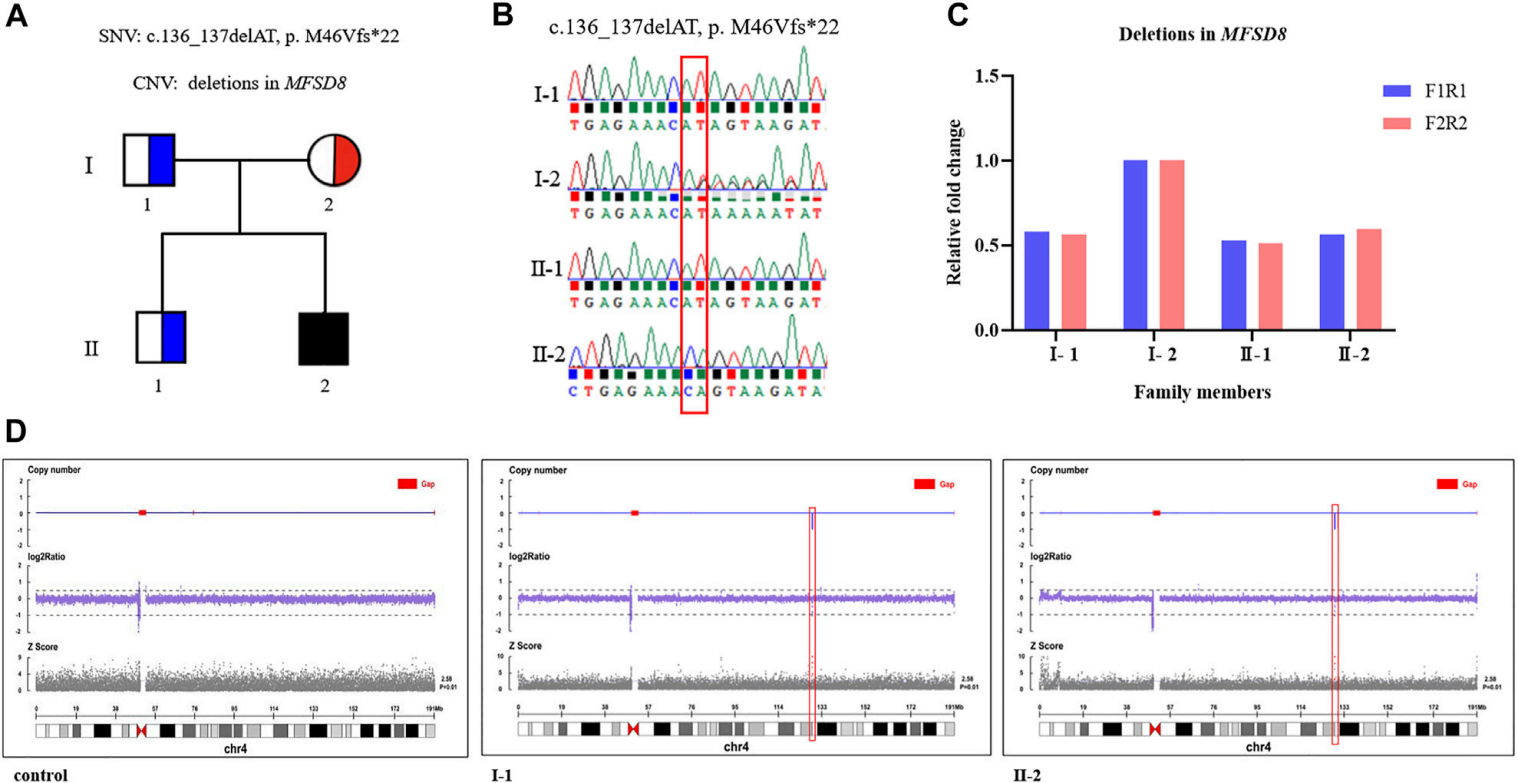
Compound heterozygous variants were identified by WES and CNV-seq. **(A)** A Chinese family with CLN7 and *MFSD8* mutations. The black filled-in square represents the proband (c.136_137delAT, p. M46Vfs*22, and whole *MFSD8* deletion), the blue half-filled squares represent the heterozygous carriers of the *MFSD8* deletions, and the red half-filled circle represents the heterozygous carrier of a novel two-nucleotide deletion (c.136_137delAT, p. M46Vfs*22). **(B)** Sanger sequencing validated the c.136_137delAT (p. M46Vfs*22) variant in the family. **(C)** Deletions in *MFSD8* were confirmed by qPCR. **(D)** CNV-seq detected an ∼130 kb deletion at 4q28.2 in both the father and proband.

The average coverage for exome sequencing of the proband was approximately 100×, and the mean sequencing depth of the deletion region (chr4:128846736 -128976763) at 4q28.2 was 46×, respectively. After a step-by-step bioinformatics analysis containing base calling, variant annotation, and biological function prediction, only a novel homozygous mutation in *MFSD8* (NM_001371596.2: c.136_137delAT, p. M46Vfs*22) was identified as being responsible for the boy’s phenotype. The AT deletion caused a frameshift mutation with a change in amino acids from 46 to 68 and introduced a new terminating TAA codon at position 69. According to ACMG guidelines, the c.136_137delAT (p. M46Vfs*22) variant is classified as “likely pathogenic (LP)” (one PVS1: LOF mutation + one PM2: the frequency of all normal population databases is less than 0.0005). Sanger sequencing revealed that the mother was a heterozygous c.136_137delAT carrier and that the father was a non-carrier (Figure 2). These data support the view that one mutation was inherited from the mother and another was *de novo*. To further investigate whether the proband’s GDD phenotype was related to the deletion of the paternal genome, we performed qPCR using primers to amplify exon 11—intron 11 (F1R1) and exon 12 (F2R2) of *MFSD8* (NM_001371596.2). Deletions were validated in the proband and his father. Furthermore, we conducted CNV-seq and detected a deletion of approximately 130 kb (chr4:128846736–128976763) at 4q28.2 and confirmed that the identified deletion was paternal, indicating that both the father and proband were heterozygous for the whole *MFSD8* deletion (Figure 2). As far as we know, the identified ∼130 kb deletion is not present in our homemade exome database, which contains 200 samples, or in public databases. In addition, according to the ACMG guidelines for sequence variants, the deletion was classified as “pathogenic”. All of the above results indicated that the compound heterozygous variants were very likely to be the genetic cause of the proband’s GDD phenotype.

## Discussion

GDD is an underdiagnosed condition, likely due to its heterogeneous etiology, presentation, and course (Vasudevan and Suri, 2017; Belanger and Caron, 2018). CLN7 is initially diagnosed as GDD and remains a devastating pediatric disease for which there are currently no cures (McBride et al., 2018; Kohlschutter et al., 2019). Although treatments may be useful to slow or even halt disease progression, few therapies are unlikely to reverse the disease (Specchio et al., 2021). In this work, we identified a 4-year-old boy with CLN7 harboring compound heterozygous mutations in *MFSD8* (c.136_137delAT and one whole gene deletion).

**FIGURE 3:**
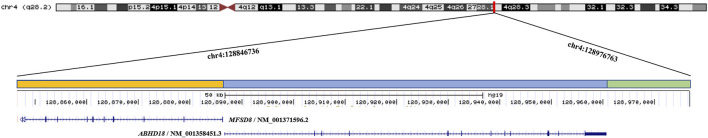
The ∼130 kb (chr4:128846736 - 128976763) deletion identified by CNV-seq extends from *MFSD8* to *ABHD18* at 4q28.2 in the proband. The orange bar represents the deletion region of *MFSD8*, the blue bar represents the deletion region of *ABHD18*, and the green bar indicates the deletion region without any known gene.

The etiology of CLN7 was due to mutations in the *MFSD8* gene. As a member of the major facilitator superfamily of active transport proteins, the MFSD8 protein posits transport solutes across the lysosomal membrane to aid in the digestion and clearance of cellular biomolecules, although the exact function of MFSD8 remains elusive (Sharifi et al., 2010). Human Gene Mutation database (HGMD), indicates that more than 40 mutations have been reported in association with CLN7, among which 30 mutations are nonsense or missense, five mutations are splicing, while six are small indel mutations. Because CLN7 exhibits a variable spectrum of clinical manifestations and the genetic variants may have different effects on the presence and function of the MFSD8, the explanation for the phenotypic variability most likely resides in the genotype of the patients. Non-functional variants result in severe clinical features of CLN7 while dysfunctional variants with residual activity may result in a markedly attenuated presentation. To the best of our knowledge, our case is the first report describing an ∼130 kb deletion at 4q28.2 in a Chinese patient with CLN7. The deleted region contained *MFSD8* and *ABHD18* genes (Figure 3). Despite the ubiquitous expression of *ABHD18*, the characteristics and function of *ABHD18* are still unknown. *MFSD8* is the causative gene of CLN7, which supports the conclusion that the severe phenotype seen in this patient was attributable to biallelic null variants leading to non-production of MFSD8. This finding will give us a better understanding of the pathogenicity of *MFSD8* and provide new insight into how *MFSD8* works and functions to cause CLN7.

*MFSD8* plays an important role during growth and development, not only in humans but also in other animals. In fact, animals such as Japanese macaques and mice with mutations in the *MFSD8* gene had similar symptoms to humans with *MFSD8* mutations. Japanese macaques with a homozygous frameshift mutation in the *MFSD*8 gene (*MFSD8* ^−/−^) display progressive neurological deficits, including visual impairment, tremors, incoordination, ataxia and impaired balance (McBride et al., 2018). Homozygous *MFSD8* (tm1a/tm1a) mice biochemically resembled human CLN7 patients, showing signs of the accumulation of autofluorescent material in the brain and peripheral tissues (Damme et al., 2014).

NCLs are characterized by decreased cognitive impairment, motor deterioration, progressive visual loss, and uncontrolled seizures (Mole et al., 2005). Our case carried two severe trans-mutations (one frameshift deletion and one whole gene deletion) without ocular abnormalities, which provides more evidence for the genotype-phenotype model in which a combination of a severe and mild variant cause nonsyndromic macular dystrophy with central cone involvement, and two severe mutations cause variant late-infantile neuronal ceroid lipofuscinosis (Roosing et al., 2015).

Autosomal recessive disorders such as CLN7 have been associated with homozygous or compound heterozygous mutations. In our report, no definite diagnosis could be given to the 4-year-old boy with GDD until WES was conducted. Initially, when focusing only on the c.136_137delAT (p. M46Vfs*22), the two-nucleotide deletion was thought to be homozygous, which seemed to fit the recessive inheritance pattern in consanguineous families. However, this conclusion was determined to be incorrect when the deletions were verified by qPCR. This uncommon compound heterozygous mutation composed of one two-nucleotide deletion inherited from the mother and one large mutation inherited from the father reminds us of the important role of WES accompanied by CNV-seq in clinical diagnosis. With the correct explanation for causing CLN7, appropriate medical genetic counseling can be given to this family. Therefore, for patients exhibiting homozygous mutations in *MFSD8*, medical staff should focus on the whole *MFSD8* gene deletion.

In summary, the clinical manifestation of movement disorders in patients with CLN7 has not been fully elucidated. Here, whole *MFSD8* gene deletion has been found to underlie CLN7. The uncommon compound heterozygous *MFSD8* mutations in this family show further heterogeneity of CLN7 at the DNA level. The increasing recognition of different mutations may provide insight into the unknown mechanisms involved in the development of CLN7.

## Data Availability

The datasets presented in this study can be found in online repositories. The names of the repository/repositories and accession number(s) can be found in the article/Supplementary Material.
