# Supplementary material for: Case Report: Novel MFSD8 Variants in a Chinese Family With Neuronal Ceroid Lipofuscinoses 7
Source: Front Genet. 2022 Jan 26;13:807515. doi: 10.3389/fgene.2022.807515 (PMC8826235; doi:10.3389/fgene.2022.807515)
Supplement: Supplementary file 1 [file Table1.docx]

**Table S1. Primers for Amplify DNA Sequences in *MFSD8***

| **Gene name** | **Primer sequence** | | **length** |  |  |
| --- | --- | --- | --- | --- | --- |
| ***MFSD8*** | F1: 5’-AATCCCTCAAATCAGTCTGTGT  R1: 5’-GGCTATCCAGTCTGCAATCT | | 170 |  |  |
| ***MFSD8*** | F2: 5’-GCTTTAGACCAGGAAGTGCC  R2: 5’- ATTCAGCCTGGTGTGTGGA | | 162 |  |  |
| ***ALB*** | | | F: 5’- AGTGCACTTGTTGAGCTCGTG  R: 5’- GCAAAGCAGGTCTCCTTATCG | 128 | |
